# Low-level viremia episodes appear to affect the provirus composition of the circulating cellular HIV reservoir during antiretroviral therapy

**DOI:** 10.3389/fmicb.2024.1376144

**Published:** 2024-05-22

**Authors:** Xiao Sun, Hui Zhang, Xiangchen Kong, Nan Li, Tong Zhang, Minghui An, Haibo Ding, Hong Shang, Xiaoxu Han

**Affiliations:** ^1^State Key Laboratory for Diagnosis and Treatment of Infectious Diseases, National Health Commission (NHC) Key Laboratory of AIDS Prevention and Treatment, National Clinical Research Center for Laboratory Medicine, The First Hospital of China Medical University, China Medical University, Shenyang, China; ^2^Collaborative Innovation Center for Diagnosis and Treatment of Infectious Diseases, Hangzhou, China; ^3^Clinical Laboratory, Shenyang Women’s and Children’s Hospital, Shenyang, China

**Keywords:** HIV-1, low-level viremia, HIV-1 reservoir, diversity, divergence

## Abstract

Low-level viremia (LLV) ranging from 50 to 1,000 copies/ml is common in most HIV-1-infected patients receiving antiretroviral therapy (ART). However, the source of LLV and the impact of LLV on the HIV-1 reservoir during ART remain uncertain. We hypothesized that LLV may arise from the HIV reservoir and its occurrence affect the composition of the reservoir after LLV episodes. Accordingly, we investigated the genetic linkage of sequences obtained from plasma at LLV and pre-ART time points and from peripheral blood mononuclear cells (PBMCs) at pre-ART, pre-LLV, LLV, and post-LLV time points. We found that LLV sequences were populated with a predominant viral quasispecies that accounted for 67.29%∼100% of all sequences. Two episodes of LLV in subject 1, spaced 6 months apart, appeared to have originated from the stochastic reactivation of latently HIV-1-infected cells. Moreover, 3.77% of pre-ART plasma sequences were identical to 67.29% of LLV-3 plasma sequences in subject 1, suggesting that LLV may have arisen from a subset of cells that were infected before ART was initiated. No direct evidence of sequence linkage was found between LLV viruses and circulating cellular reservoirs in all subjects. The reservoir size, diversity, and divergence of the PBMC DNA did not differ significantly between the pre- and post-LLV sampling points (*P* > 0.05), but the composition of viral reservoir quasispecies shifted markedly before and after LLV episodes. Indeed, subjects with LLV had a higher total PBMC DNA level, greater viral diversity, a lower proportion of variants with identical sequences detected at two or more time points, and a shorter variant duration during ART compared with subjects without LLV. Overall, our findings suggested that LLV viruses may stem from an unidentified source other than circulating cellular reservoirs. LLV episodes may introduce great complexity into the HIV reservoir, which brings challenges to the development of treatment strategies.

## Introduction

Antiretroviral therapy (ART) can effectively suppress HIV replication, thereby greatly improving the quality of life and reducing the fatality rate among infected patients (Wandeler et al., 2016). However, ART does not eradicate HIV infection due to the persistence of viral reservoirs. Long-lived reservoirs of infected CD4^+^ T cells that carry replication-competent HIV-1 can rapidly lead to viral rebound if ART is interrupted (De Scheerder et al., 2019). Recent study has shown that prolonged cellular lifespan and the clonal expansion of HIV-infected cells are primarily responsible for the maintenance of the HIV reservoir (Reeves et al., 2018). Moreover, ongoing HIV replication, especially in specific anatomic locations such as the lymph node, gut-associated lymphoid tissue, and cerebrospinal fluid, was reported to continuously replenish the HIV reservoir (Lorenzo-Redondo et al., 2016). Consequently, an improved understanding of the mechanisms underlying HIV persistence in reservoirs during ART will contribute to the development of HIV eradication strategies.

Although most proviruses in the HIV-1 reservoir remain latent during ART, a small fraction is transcriptionally active, leading to low levels of viremia. Residual viremia (RV) with viral loads (VLs) below 50 copies/ml was reported to contribute to rebound viremia following treatment interruption (Aamer et al., 2020). Notably, several studies have demonstrated that low-level viremia (LLV) ranging from 50 to 1,000 copies/ml is present in ∼30% of HIV-1-infected subjects receiving ART (Santoro et al., 2014; World Health Organization, 2016; Zhang et al., 2020). LLV episodes may contribute to overall immune activation, high HIV-1 transcriptional activity (Karlsson et al., 2004; Han et al., 2020), microbial translocation and inflammation (Reus et al., 2013; Hofstra et al., 2014). Like with RV (Aamer et al., 2020; Halvas et al., 2020; White et al., 2023), identifying the source of LLV may increase the understanding of HIV reservoirs capable of producing infectious virions during ART, provide clues for rebound viremia, and potentiate the development of strategies for eliminating or reducing the HIV reservoir.

The origin of LLV and the impact of LLV on HIV reservoirs remain incompletely understood. Viral sequences in LLV were reported to be similar to those found in the early stages of HIV infection, supporting that the activation of latently infected cells may be the source of LLV (Nettles et al., 2005), especially regarding the clonal expansion of reservoir cells (Tobin et al., 2005; Vancoillie et al., 2017; Bull et al., 2018). In addition, through genetic analysis, an accumulation of new drug-resistance mutations and viral evolution were found in LLV viral sequences, which suggested that viral replication was ongoing (Tobin et al., 2005). Importantly, patients with LLV were reported to have significantly larger HIV-1 reservoirs and higher RNA transcript levels compared with those without LLV (Bachmann et al., 2019; Suzuki et al., 2021). However, whether LLV viruses replenish HIV reservoirs remains unclear. Elucidating the source of LLV and the impact of LLV on the reservoir is expected to inform our understanding of the mechanisms involved in HIV persistence.

In this study, we specifically aimed to reveal the genetic linkage of LLV sequences with sequences detected at pre-ART, pre-LLV, and post-LLV time points to understand the source of LLV and determine whether LLV viruses replenish the HIV reservoir. Additionally, we compared the changes in viral composition occurring in circulating cellular HIV reservoirs between subjects with and without LLV.

## Materials and methods

### Study subjects

This was a retrospective study based on a prospectively followed cohort of HIV-1-infected patients from the First Affiliated Hospital of China Medical University in Shenyang, Liaoning Province, China. The criteria used for the selection of subjects with LLV in this study included (1) persistent LLV, namely, at least two consecutive episodes of LLV with a VL of 50–1,000 copies/ml after ART; (2) follow-up visits every 6 months; (3) multiple specimens, including plasma obtained at pre-ART and LLV time points, and peripheral blood mononuclear cell (PBMC) samples acquired at pre-ART, pre-LLV, LLV, and post-LLV time points; and (4) no change of treatment regimen for HIV infection during the LLV period. Subjects without LLV were included based on the following criteria: (1) achieving viral suppression (undetectable VL) after receiving ART and maintaining it for at least 2 years, (2) follow-up visits every 6 months, and (3) available PBMC specimens spanning 2–4 time points. Four subjects with LLV and four without LLV were recruited for the study. Plasma was stored at −80°C. PBMCs were separated by Ficoll-Paque Plus (GE Healthcare Bio-Sciences, USA) density gradient centrifugation and cryopreserved in liquid nitrogen. The study was approved by the Ethics Committee of the First Affiliated Hospital of China Medical University ([2021]–161). All study participants signed informed consent forms.

### Quantification of total HIV–1 DNA

The levels of total HIV-1 DNA in PBMCs were quantified by droplet digital polymerase chain reaction (ddPCR, Bio-Rad). Real-time PCR was carried out as duplex reactions using primers and fluorescent probes designed to target the HIV-1 5′ LTR/gag gene (HXB2: 684–810) and the housekeeping gene RPP30. Primers and probes, as well as PCR program were described previously (Jiang et al., 2020). The number of droplets was counted using the QX200 droplet reader (Bio-Rad). Wells with >10,000 droplets were analyzed by QuantaSoft software (Bio-Rad). The normalized HIV-1 DNA copies were expressed as log_10_ HIV DNA/million PBMCs. CD4^+^ T-cell counts and HIV VLs were determined as previously described (Zhang et al., 2015, 2022).

### Nucleic acid extraction and amplification

When plasma with VLs ranging from 50 to 1,000 copies/ml, virus particles from 1-ml aliquots of plasma were pelleted by ultracentrifugation at 100,000 × *g* for 2.5 h at 4°C, with removal of all but 140 μl of the plasma over the pellet. RNA was extracted from plasma using the QIAamp Viral RNA Mini Kit (Qiagen, Germany) and from PBMCs using the RNeasy Plus Mini Kit (Qiagen, Germany). The RNA was reverse-transcribed into cDNA using the Transcriptor First Strand cDNA Synthesis Kit (Roche, Germany) with primer Rev2-1 (HXB2: 5957–5980). Total HIV-1 DNA was extracted from PBMCs using the QIAamp DNA Mini Kit (Qiagen, Germany).

The *pol* fragment was amplified by nested PCR using the KOD-Plus-Neo High-fidelity polymerase (TOYOBO LIFE SCIENCE, Japan). The first round of PCR was performed with the outer primers MAW26 (HXB2: 2029–2050) and RT21 (HXB2: 3509–3539) and the second round was performed using the inner primers 3-2F (HXB2: 2583–2605) and 3-2R (HXB2: 2997–3016). Nested PCR was conducted with the following cycling parameters: 94°C for 2 min, followed by 40 cycles of 98°C for 10 s, 58°C for 30 s, and 68°C for 1.5 min, with a final extension step of 68°C for 10 min.

### Deep sequencing and phylogenetic analysis

Positive amplification products were purified with Agencourt AMPure XP beads (Beckman Coulter, USA), quantified using the Qubit dsDNA BR Assay Kit (Life Technologies, USA), and then sequenced on the Illumina MiSeq platform at Sangon Biotech, China. The raw data were analyzed employing Geneious software (2021.2.2). Sequences were trimmed, and reads with a *Q*-score greater than 30 were retained. Sequences that were 100% identical were grouped to form consensus sequences. HIV-1 quasispecies were defined as consensus sequences appearing more than 100 times and were included in the analysis (Giatsou et al., 2020). Sequences were aligned using HIVAlign and were edited with Bioedit software.

Phylogenetic trees were generated in FastTree v.2.1.9 using the maximum-likelihood (ML) method, with the GTR + G + I substitution model. The tree was edited in FigTree v.1.4.3. Diversity and divergence over time were calculated using Mega software (version 7). For divergence, the distance of sequences obtained at a given time point was measured relative to those of the most recent common ancestor, which were determined based on viruses from pre-ART samples using Mega software (version 7).

### Statistical analyses

Statistical analysis was performed using GraphPad Prism v.9.0.2 (GraphPad Software, San Diego, CA, USA). Wilcoxon matched pairs signed rank tests were used to compare differences in reservoir size, diversity, and divergence between pre-LLV and post-LLV time points. The size of the reservoir, the diversity of PBMC HIV DNA, and the proportion of variants in subjects with and without LLV were compared using Mann–Whitney tests. A *P*-value of less than 0.05 was considered significant.

## Results

### Clinical characteristics of subjects in this study

**FIGURE 1:**
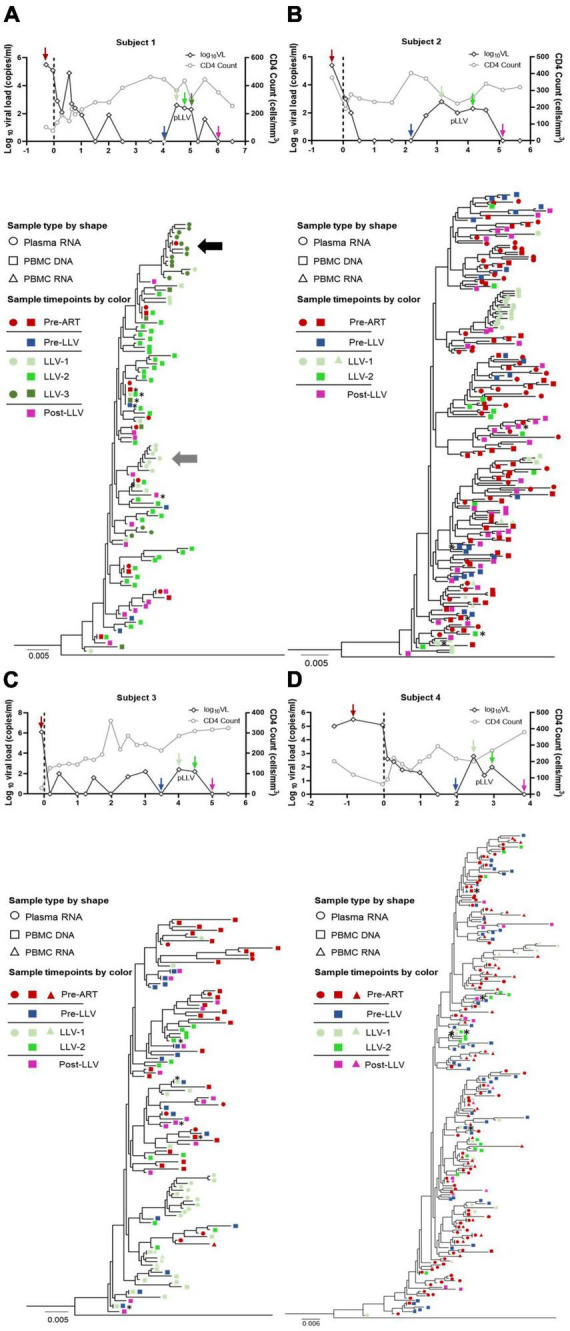
Clinical data and phylogenetic tree for subjects with LLV. HIV-1 phylogenetic trees and longitudinal clinical data relating to VLs (black hollow diamonds), CD4^+^ T-cell counts (gray hollow circles), and years post-ART (*x*-axis) for subjects 1, 2, 3, and 4 (**A–D**, respectively). The time points selected for deep sequencing of the HIV-1 *pol* gene from plasma and PBMCs have different color of arrows, which are consistent with the phylogenetic tree. Maximum-likelihood within-host phylogenetic trees were rooted using CRF01_AE (accession number, AF197340). Viral sequences are represented by circles (plasma RNA), squares (PBMC DNA), and triangles (PBMC RNA). The key indicates the color code for the time points when specimens were collected [pre-ART (red), pre-LLV (blue), LLV (green), and post-LLV (pink)]. The two clusters containing the most virus quasispecies induced from LLV-1 and LLV-3 are shown with a horizontal gray arrow and a black arrow, respectively. The dominant quasispecies of PBMC DNA at each time point is denoted by an asterisk (*).

In total, four subjects with and four without LLV were included in this study (Table 1). All subjects were male and were infected with HIV-1 through homosexual contacts. The median age at HIV diagnosis, median VL, and CD4^+^ T-cell count at ART initiation did not differ significantly between subjects with and those without LLV (*P* > 0.05). The suppression of plasma HIV VL to <50 copies/ml was observed at 11.94 (IQR, 3.09–18) months after ART initiation for subjects with LLV and at 2.28 (IQR, 1.08–10.50) months after ART initiation for subjects without LLV. The duration of persistent LLV was 23.10 (IQR, 19.26–32.43) months. During persistent LLV, the median VL was 2.32 (IQR, 2.03–2.49) log_10_ copies/ml and the median CD4^+^ T-cell count was 295 (IQR, 261–360) cells/μl. Sequences obtained at the time points indicated in Figure 1 and Supplementary Figure 1. The number of raw reads was not significantly different between subjects with and those without LLV (*P* > 0.05). The median number of quasispecies after ART for the PBMCs was 13 (IQR, 9–21) for subjects with LLV and 15 (IQR, 13–17) for those without LLV.

**TABLE 1:** Clinical characteristics of HIV-1-infected subjects with and without LLV.

Characteristic	Subjects with LLV (*n* = 4)	Subjects without LLV (*n* = 4)	*P*-value
Gender	Male	Male	NA
Age at HIV diagnosis (years), M (IQR)	36 (31–47)	28 (24–31)	0.057
CD4^+^ T cells at ART initiation (cells/μl), M (IQR)	70 (37–302)	184 (60–333)	0.771
Log_10_ VL at ART initiation (copies/ml), M (IQR)	5.29 (5.09–5.93)	4.19 (3.46–5.30)	0.200
Months from ART initiation to suppression of VL to <50 copies/ml, M (IQR)	11.94 (3.09–18.00)	2.28 (1.08–10.50)	0.200
CD4^+^T cells at LLV episodes (cells/μl), M (IQR)	295 (261–360)	NA	NA
Log_10_ VL at LLV episodes (copies/ml), M (IQR)	2.32 (2.03–2.49)	NA	NA
LLV duration (months), M (IQR)	23.10 (19.26–32.43)	NA	NA

LLV, low level of viremia; M, median; IQR, interquartile range; NA, not available.

### LLV viruses were populated with a predominant viral quasispecies

**FIGURE 2:**
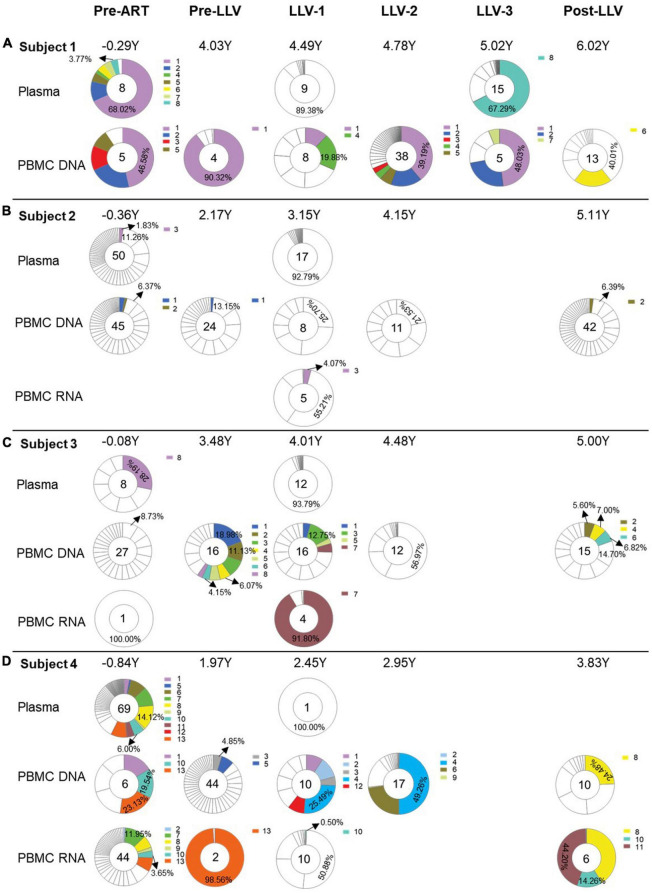
Longitudinal analysis of the proportions of quasispecies from subjects with LLV. Pie charts showing the distribution of quasispecies sequences from plasma, PBMC DNA, and PBMC RNA collected at pre-ART initiation, pre-LLV, LLV, and post-LLV time points from subjects 1 **(A)**, 2 **(B)**, 3 **(C)**, and 4 **(D)**. Years post-ART for each time point are listed at the top of the pie charts. Numbers in the center of the circles represent the total number of quasispecies. White areas in the pie chart represent sequences obtained once during follow-up. Identical sequences that were detected at two or more time points were defined as a variant. Different variants are denoted by different colors and the proportions of dominant quasispecies are listed.

To examine the characteristics of LLV viruses, a ML tree of sequences from plasma and PBMC samples collected at pre-ART, pre-LLV, LLV, and post-LLV time points was constructed for each subject (Figures 1A–D). For subject 1, LLV plasma sequences were obtained for two time points with an interval of approximately 6 months. Nine quasispecies were identified for LLV-1 and 15 for LLV-3 (Figures 1A, 2A). A total of 17 quasispecies from subject 2 (Figures 1B, 2B) and 12 from subject 3 (Figures 1C, 2C) were present in a single cluster in the phylogenetic tree. The LLV viruses were composed of multiple quasispecies (median number: 14 [IQR, 10–17]) but were populated with a predominant quasispecies that accounted for 67.29%∼93.79% of all the sequences in subjects 1–3 (Figures 2A–C). Meanwhile, the LLV from subject 4 represented only a quasispecies (Figure 2D). Overall, LLV viruses were populated with a predominant viral quasispecies.

For subject 1, LLV sequences from LLV-1 and LLV-3 exhibited a dispersed distribution across the ML tree and displayed obvious topological differences (Figure 1A). Clearly distinct clades were observed between 91.28% of sequences (near the gray arrow in Figure 1A) obtained from LLV-1 and 78.19% of sequences (near the black arrow in Figure 1A) derived from LLV-3. Only one quasispecies accounting for 0.61% of sequences from LLV-1 and one quasispecies accounting for 0.75% of sequences from LLV-3 presented on the same node (Figure 1A). The two sets of LLV appeared to have originated from the stochastic reactivation of different latently HIV-1-infected cells.

### There was limited relatedness between LLV viruses and circulating cellular reservoirs

To identify the source of LLV, we further analyzed the relationship between LLV sequences and sequences detected at pre-ART and pre-LLV time points. In subject 1, a total of 3.77% of pre-ART plasma sequences were identical to 67.29% of LLV-3 plasma sequences (Figures 1A, 2A, variant 8), suggesting that this population could be a source of LLV. In contrast, the LLV sequences detected in subjects 2, 3, and 4 did not match sequences obtained from pre-ART plasma or PBMC DNA (Figures 1B–D, 2B–D). No direct evidence of sequence linkage was detected between LLV viruses and circulating cellular reservoirs.

Actively transcribed intracellular HIV RNA was reported to predict the rebound viruses (Kearney et al., 2015). Accordingly, we next analyzed whether there was an association between pre-LLV PBMC RNA and LLV viruses, however, no identical sequences were detected between the two groups in subject 2 (Figure 2B) or 4 (Figure 2D). In addition, we found that 0.50% of LLV-1 PBMC RNA sequences were identical to 6.00% of pre-ART plasma sequences, 19.54% of pre-ART PBMC DNA sequences, 3.65% of pre-ART PBMC RNA sequences, and 14.26% of post-LLV PBMC RNA sequences in subject 4 (variant 10, Figure 2D). The transcriptionally active cell population were infected before ART initiation, or their descendants, presumably expanded and persisted during ART for at least 4.67 years. Similarly, 4.07% of LLV-1 PBMC RNA sequences were identical to 1.83% of pre-ART plasma sequences from subject 2, separated by an interval of 3.51 years (variant 3, Figure 2B). The expanded, transcriptionally active cells persisted during ART, but did not lead to LLV episodes. Taken together, these results suggested that there was limited relatedness between LLV viruses and circulating cellular reservoirs.

### LLV viruses were not detected to replenish into the circulating cellular HIV reservoirs

**FIGURE 3:**
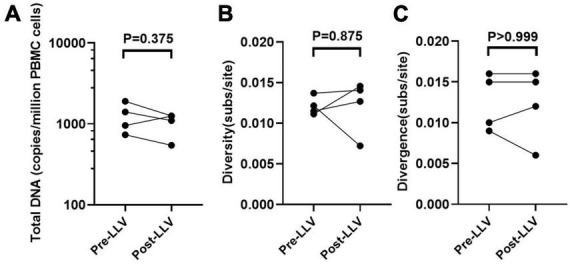
The impact of LLV episodes on the HIV reservoir. The level (the normalized HIV-1 DNA copies are expressed as log_10_ HIV DNA/million PBMCs) **(A)**, diversity **(B)**, and divergence **(C)** of total PBMC DNA were compared at time points before and after LLV.

To further detect the impact of LLV on HIV reservoirs, we compared the size and composition of the viral reservoir populations between pre- and post-LLV time points. We found that LLV episodes did not influence the total HIV DNA level, with a median of 1,179 copies/million PBMCs (IQR, 789–1779) at the pre-LLV time point compared with 1,172 copies/million (IQR, 683–1255) at the post-LLV time point (*P* = 0.375; Figure 3A). Moreover, the detected LLV sequences did not match post-LLV PBMC DNA sequences for any of the subjects (Figures 1, 2), indicative of no replenishment of LLV viruses into circulating cellular HIV reservoirs. In addition, in subject 3, 21.35% (731/3,424) sequences at pre-LLV timepoint were identical to 19.42% (638/3,285) sequences at post-LLV timepoint (Figure 2C). For subjects 1, 2, and 4, meanwhile, no pre-LLV PBMC DNA sequences were identical to post-LLV sequences, with intervals of 1.99, 2.94, and 1.86 years, respectively (Figures 2A, B, D), suggesting that the composition of quasispecies derived from HIV reservoirs fluctuated significantly before and after LLV. Surprisingly, neither the diversity nor the divergence of the PBMC DNA differed significantly between the pre- and post-LLV sampling points (*P* = 0.875 and *P* > 0.999, respectively; Figures 3B, C).

### Dynamic changes in variant composition were detected in circulating HIV reservoirs in subjects with LLV

**FIGURE 4:**
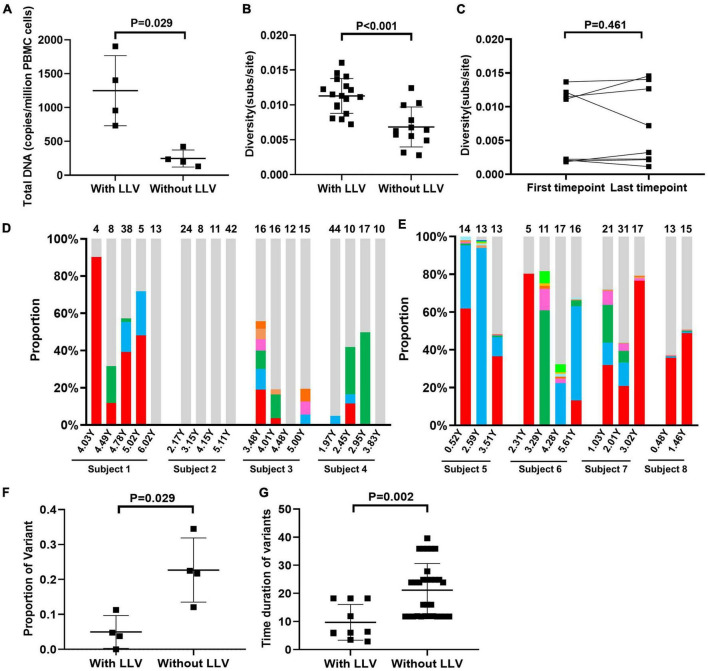
Dynamic changes in variant composition in the reservoirs of subjects with LLV. A comparative analysis of total PBMC DNA levels (the normalized HIV-1 DNA copies are expressed as log_10_ HIV DNA/million PBMCs) at the first available time point for a valid measurement of the HIV reservoir size after ART initiation **(A)** and viral diversity across the 2–4 time points during ART **(B)** between subjects with and those without LLV. **(C)** Comparison of viral diversity in PBMC DNA between the first available time point after ART initiation and the last follow-up. The proportions of each variant that was detected at two or more time points during ART in subjects with LLV **(D)** and those without LLV **(E)** are shown in histograms. The variants are denoted by different colors. Quasispecies detected at only one time point are shown in light gray. The numbers of quasispecies are listed at the top of the column. Comparison of the proportion **(F)** and persistence **(G)** of variants between subjects with and those without LLV.

As mentioned above, the composition of circulating cellular HIV reservoirs changed at post-LLV timepoint. Thus, we further compared the characteristics of viral sequences derived from HIV reservoirs between subjects with and those without LLV. The median total HIV DNA level in subjects with LLV was significantly higher than that in subjects without LLV at the pre-ART (8,300 [IQR, 7,551–9,146] vs. 391 [IQR, 208–595] copies/million PBMCs, *P* = 0.029; Supplementary Figure 3A) and at the first timepoint suitable for a valid measurement of HIV reservoir size after ART initiation (1,179 [IQR, 789–1,779] copies/million PBMCs at 2.83 [IQR, 2.02–3.89] years after ART initiation vs. 217 [IQR, 148–376] copies/million PBMCs at 2.76 [IQR, 1.60–4.099] years after ART initiation, *P* = 0.029; Figure 4A). Meanwhile, subjects with LLV showed no difference in pre-ART PBMC DNA diversity (0.0118 [IQR, 0.0062–0.0174] vs. 0.0070 [(IQR, 0.0027–0.0115] subs/site, *P* = 0.343; Supplementary Figure 3B), but exhibited greater diversity in viral sequences from HIV reservoirs across time points 2–4 compared with subjects without LLV (0.011 [IQR, 0.009–0.013] vs. 0.006 [IQR, 0.005–0.009], *P* < 0.001; Figure 4B). However, viral diversity, at 0.007 (IQR, 0.002–0.012) at the first available time point after ART initiation versus 0.005 (IQR, 0.002–0.014) at the time of the last follow-up (*P* = 0.461; Figure 4C), has remained unchanged for a median of 1.99 years (IQR, 1.61–2.98).

PBMC DNA with identical *pol* sequences that were detected at two or more time points were defined as variants. In LLV subjects 1–4, respectively 4.76% (3/63), 0% (0/85), 11.32% (6/53), and 3.85% (3/78) of variants with identical sequences were detected in PBMC DNA over a median of 6.24 (IQR, 5.16–19.76) months (Figure 4D). Interestingly, variant 1 in subject 1 was detected as a dominant quasispecies with a frequency of 43.61% (IQR, 18.64%–79.75%) across the four time points evaluated, and lasted for approximately 11.88 months (Figure 4D), indicative of its persistence. However, we are unable to distinguish that the variant is from cells infected with genetically identical viruses, the clonal expansion of HIV reservoir or both, due to the lack of HIV integration site analysis. In subjects 2, 3, and 4, meanwhile, dominant PBMC DNA quasispecies exhibited a dispersed distribution in the phylogenetic tree (Figures 1B–D). The proportion of unique quasispecies fluctuated significantly over time. In subject 2, all unique quasispecies were present at all time points (Figure 4D). In subjects without LLV (5–8), variants with identical sequences accounted for 34.48% (10/29), 22.50% (9/40), 12.07% (7/58), and 21.74% (5/23), respectively, of all the quasispecies observed over a median of 23.88 (IQR, 11.76–24.84) months (Supplementary Figure 2 and Figure 4E). The proportion of variants was significantly lower in subjects with LLV than in those without LLV (4.30% [IQR, 0.95%–9.68%] vs. 22.10% [IQR, 14.50%–31.50%], *P* = 0.029; Figure 4F). Meanwhile, variant persistence was shorter in subjects with LLV than in those without LLV (6.18 [5.19–18.24] months vs. 23.88 [11.76–24.84] months, *P* = 0.002; Figure 4G) over a follow-up period of 4.47 (IQR, 3.05–5.58) years after ART initiation.

## Discussion

In this study, we systematically investigated the source of LLV and the impact of LLV episodes on HIV-1 reservoirs. Our results revealed several notable observations, as follows: (1) LLV was populated with a predominant quasispecies and showed limited relatedness to circulating cellular reservoirs. LLV viruses detected at two consecutive time points may arise from the stochastic reactivation of the different latently HIV-1-infected cells; (2) LLV viruses did not replenish circulating cellular HIV reservoirs or affect the post-LLV PBMC DNA level; however, the composition of HIV reservoirs showed significant fluctuation; and (3) subjects with LLV had higher levels of total PBMC DNA, greater viral diversity, a lower proportion of variants with identical sequences detected at two or more time points, and reduced variant persistence compared with subjects without LLV during ART. Overall, our findings identified a previously undetected interplay between LLV episodes and the considerable stochastic fluctuations observed in circulating HIV reservoirs.

Multiple studies have investigated the associations between HIV reservoirs and RV, LLV, or rebound viruses after treatment interruption (Kearney et al., 2015; Lu et al., 2018; Aamer et al., 2020; Liu et al., 2020; Cole et al., 2022; Hendricks et al., 2022). Relatively few identical sequences were detected between RV, LLV, or rebound virus and replication-competent viruses from quantitative viral outgrowth assay (QVOA) (Bailey et al., 2006; Brennan et al., 2009; Anderson et al., 2011; Aamer et al., 2020), and in most subjects, rebound viruses and reservoir provirus did not show sequence linkages (Bailey et al., 2006; Lu et al., 2018). Interestingly, LLV viruses from the four subjects were populated with a dominant quasispecies. Moreover, LLV viruses from two consecutive time points were phylogenetically distinct. The shift in the LLV populations during ART may be due to the stochastic reactivation of the different cell populations, which might be driven by diverse antigens or cytokines (Conway and Coombs, 2011; Wang et al., 2018). Similar results have been observed in subjects with RV and rebound virus across two time points (Bailey et al., 2006; Anderson et al., 2011; Kearney et al., 2015; Aamer et al., 2020). Subsequently, we found that most LLV-3 sequences from subject 1 were genetically identical to a low proportion of viral quasispecies from pre-ART plasma, indicating that LLV virus may have arisen from a subset of cells that were infected prior to initiating ART and persisted by clonal expansion during ART. However, these LLV virus quasispecies were underrepresented in DNA recovered from circulating cellular reservoirs as they did not match PBMC DNA or RNA, suggesting that the LLV in this subject may have originated from a tissue reservoir or a circulating cellular reservoir that was not sampled.

Subjects with viral blips (plasma VLs between 20 and 200 copies/ml) during ART had significantly increased cell-associated HIV-1 transcriptional activity (Suzuki et al., 2021). Meanwhile, actively transcribed intracellular HIV RNA during ART was reported to be a predictor of viral rebound after treatment interruption (Li et al., 2016; Pasternak et al., 2020). Cells with identical proviruses that were transcriptionally active before ART interruption were proposed to be a source of initial rebound viremia (Kearney et al., 2015). We speculate that proviruses in cells expressing HIV RNA prior to LLV may serve as the source of LLV. However, although some cells or the descendants of cells that were infected before ART initiation in subject 4 continued to express HIV RNA during ART for at least 4.67 years (variant 10, Figure 2D), these cells did not lead to LLV episodes. This discrepancy could reflect limited sampling of infected PBMCs or the presence of anatomic reservoirs of HIV that were not sampled but may also contribute to LLV, as suggested by reports from Rothenberger et al. (2015) and Kearney et al. (2015). Additionally, full-length HIV sequencing is necessary to confirm whether the cell-associated RNA and LLV RNA sequences are defective.

Several studies have shown that viral rebound following treatment interruption does not impact HIV-1 latent reservoir size or diversity (Clarridge et al., 2018; Cohen et al., 2018; Salantes et al., 2018; Scutari et al., 2021). Additionally, phylogenetic analysis did not identify a substantial difference in the composition of reservoirs after ART interruption (Salantes et al., 2018). Meanwhile, no evidence was found to support that rebound virus enriched the replication-competent peripheral latent reservoir when ART was suspended (Salantes et al., 2018). Similarly, a study by Torres-Cornejo et al. (2014) found that cellular HIV reservoir replenishment was not affected by LLV episodes. In our study, we did not detect LLV viruses that entered into circulating cellular HIV reservoirs. However, we cannot exclude the possibility that the cell population carrying LLV viruses was small, and hence could not be detected among the most reservoir cells that carry defective proviruses (Ho et al., 2013).

Patients with LLV were reported to exhibit significantly higher level of pre-ART PBMC DNA and larger HIV-1 reservoirs (Bull et al., 2018; Bachmann et al., 2019; Suzuki et al., 2021). Pre-ART PBMC DNA levels were positively associated with post-ART PBMC DNA levels, while pre-ART PBMC DNA genetic diversity differed from provirus diversity. Provirus diversity reflects the accumulated diversity over the entire infection, potentially influenced by reservoir decay and hypermutations in proviral DNA induced by APOBEC3G/F (Tzou et al., 2020; Zeeb et al., 2024). Although no significant differences in the diversity and divergence of PBMC viral DNA were found between before and after LLV, there were obvious HIV reservoir population shifts not only across the two time points but also during the whole process of follow-up during ART. There are several potential explanations for the considerable stochastic fluctuation in the composition of HIV reservoirs in subjects with LLV. One of the most important reasons is the limited sampling depth of circulating cellular reservoirs, which only represents a small fraction of the total reservoirs. However, even under the similar conditions, subjects with LLV exhibited a lower proportion of variants and reduced variant persistence during ART compared to those without LLV. Another possibility is that subjects with LLV showed higher levels of immune activation than subjects without LLV (Ostrowski et al., 2005; Han et al., 2020), potentially accelerating the turnover of latent HIV reservoirs and facilitating the exchange of reservoirs between blood and tissues. Lymph nodes were suggested to serve as the sanctuary site for latent HIV, CD4^+^ T cells carrying latent viruses circulate between blood and lymphoid tissues, resulting in the same overall frequencies of HIV-1 provirus between these two compartments (Vibholm et al., 2019). The more clearly stochastic fluctuation in the composition of the HIV reservoirs in subjects with LLV complicates immune clearance and hampers the development of curative strategies for HIV.

Our study had several limitations. First, this was a small study with only four subjects with LLV. Further studies using a larger cohort are warranted to clarify whether our results can be extrapolated to other LLV patients. Second, the source of LLV was determined by identifying identical sequences in LLV viruses and PBMC DNA/RNA. However, the short fragment of the *pol* sequence amplified in our study was not sufficient to predict identical whole-genome sequences, leading to the possible overestimation of “identical” sequences. Furthermore, we only sampled viruses from peripheral blood, while in-depth mapping of peripheral and tissue reservoirs is required to reveal the sources of LLV.

## Conclusion

In conclusion, our results indicated that LLV may arise from a subset of cells that are infected prior to initiating ART. These cells are not persisted by viral replication and are stochastically reactivated during treatment. However, LLV viruses showed limited relatedness with circulating cellular HIV reservoirs. Subjects with LLV displayed considerable stochastic fluctuation in the composition of the respective circulating cellular reservoirs. LLV episodes may increase the complexity of HIV reservoirs. Our study supports the need to strengthen the management of LLV episodes so as to reduce the challenges associated with HIV reservoir clearance. Novel treatment strategies targeting reservoirs that contribute to LLV are needed for the treatment of HIV.

## Data availability statement

The data presented in the study are deposited in the NCBI repository, accession numbers PRJNA1072152 and PRJNA1072258.

## Ethics statement

The studies involving human participants were approved by the Ethics Committee of the First Affiliated Hospital of China Medical University ([2021]–161). The patients/participants provided their written informed consent to participate in this study.

## Author contributions

XS: Data curation, Formal analysis, Investigation, Methodology, Software, Writing – original draft, Writing – review & editing. HZ: Data curation, Formal analysis, Investigation, Methodology, Software, Writing – original draft, Writing – review & editing. XK: Data curation, Investigation, Methodology, Software, Writing – original draft. NL: Data curation, Investigation, Methodology, Software, Writing – original draft. TZ: Data curation, Investigation, Methodology, Writing – original draft. MA: Methodology, Software, Writing – original draft. HD: Data curation, Investigation, Writing – original draft. HS: Conceptualization, Funding acquisition, Validation, Visualization, Writing – review & editing, Writing – original draft. XH: Conceptualization, Validation, Visualization, Writing – review & editing, Writing – original draft.
