# Supplementary material for: Low-level viremia episodes appear to affect the provirus composition of the circulating cellular HIV reservoir during antiretroviral therapy
Source: Front Microbiol. 2024 May 22;15:1376144. doi: 10.3389/fmicb.2024.1376144 (PMC11150674; doi:10.3389/fmicb.2024.1376144)
Supplement: Supplementary file 1 [file Data_Sheet_1.zip › Supplementary materials/Supplementary Figure legends.docx]

**Supplemental Figure 1. Clinical data and HIV-1 phylogenetic tree for subjects without LLV.** HIV-1 phylogenetic trees and longitudinal clinical data for VLs (black hollow diamonds), CD4^+^ T-cell counts (grey hollow circles), and years post-ART (*x*-axis) are shown for subjects 5, 6, 7, and 8 (A, B, C, and D, respectively). The time points selected for deep sequencing of the HIV-1 *pol* gene from PBMCs after ART initiation have VL symbols of different color of arrows, which are consistent with the phylogenetic tree. Maximum-likelihood within-host phylogenetic trees were rooted using CRF01_AE (Accession number, AF197340). Viral sequences that were obtained at different time points are represented by different colors. The dominant quasispecies of PBMC DNA at each time point is denoted by an asterisk (*).

**Supplemental Figure 2. Longitudinal analysis of the proportions of quasispecies from subjects without LLV.** Pie charts showing the distribution of quasispecies sequences from PBMC DNA at different time points for subjects 5 (A), 6 (B), 7 (C), and 8 (D). Numbers in the center of the circles represent the total numbers of quasispecies. White areas in the pie chart represent sequences obtained once. Identical sequences that were detected at two or more time points were defined as a variant. Different variants are denoted by different colors and the proportions of the dominant quasispecies are listed.

**Supplemental Figure 3. Comparison of pre-ART PBMC DNA level and PBMC DNA diversity between subjects with and those without LLV.** The PBMC DNA level (A), and DNA diversity (B) at the pre-ART timepoint were compared between subjects with and those without LLV.
