# Supplementary figures and images for: Low-level viremia episodes appear to affect the provirus composition of the circulating cellular HIV reservoir during antiretroviral therapy
Source: Front Microbiol. 2024 May 22;15:1376144. doi: 10.3389/fmicb.2024.1376144 (PMC11150674; doi:10.3389/fmicb.2024.1376144)

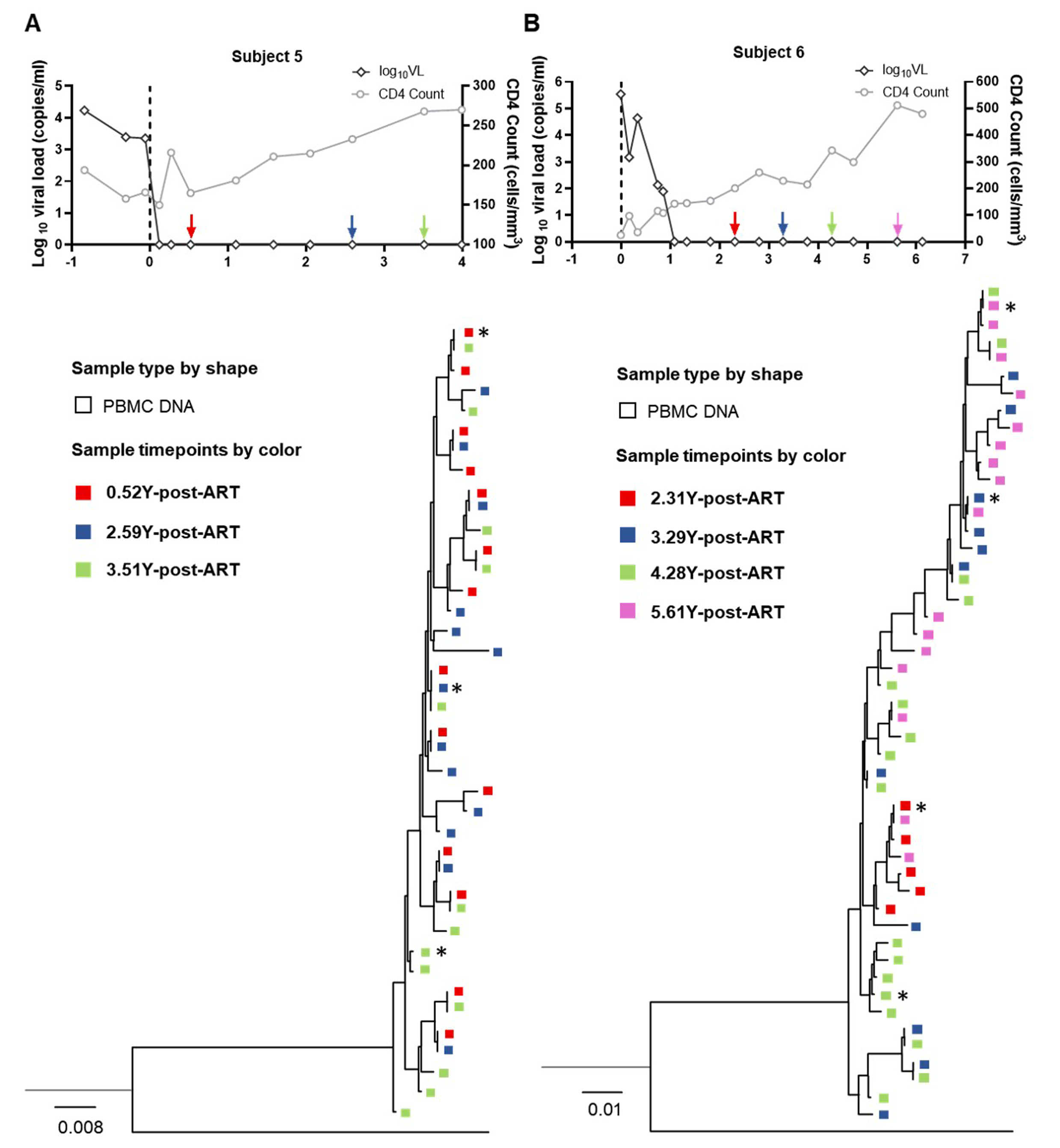

Supplement: Supplementary file 1 [file Data_Sheet_1.zip › Supplementary materials/Supplementary Figure S1-AB.tif]

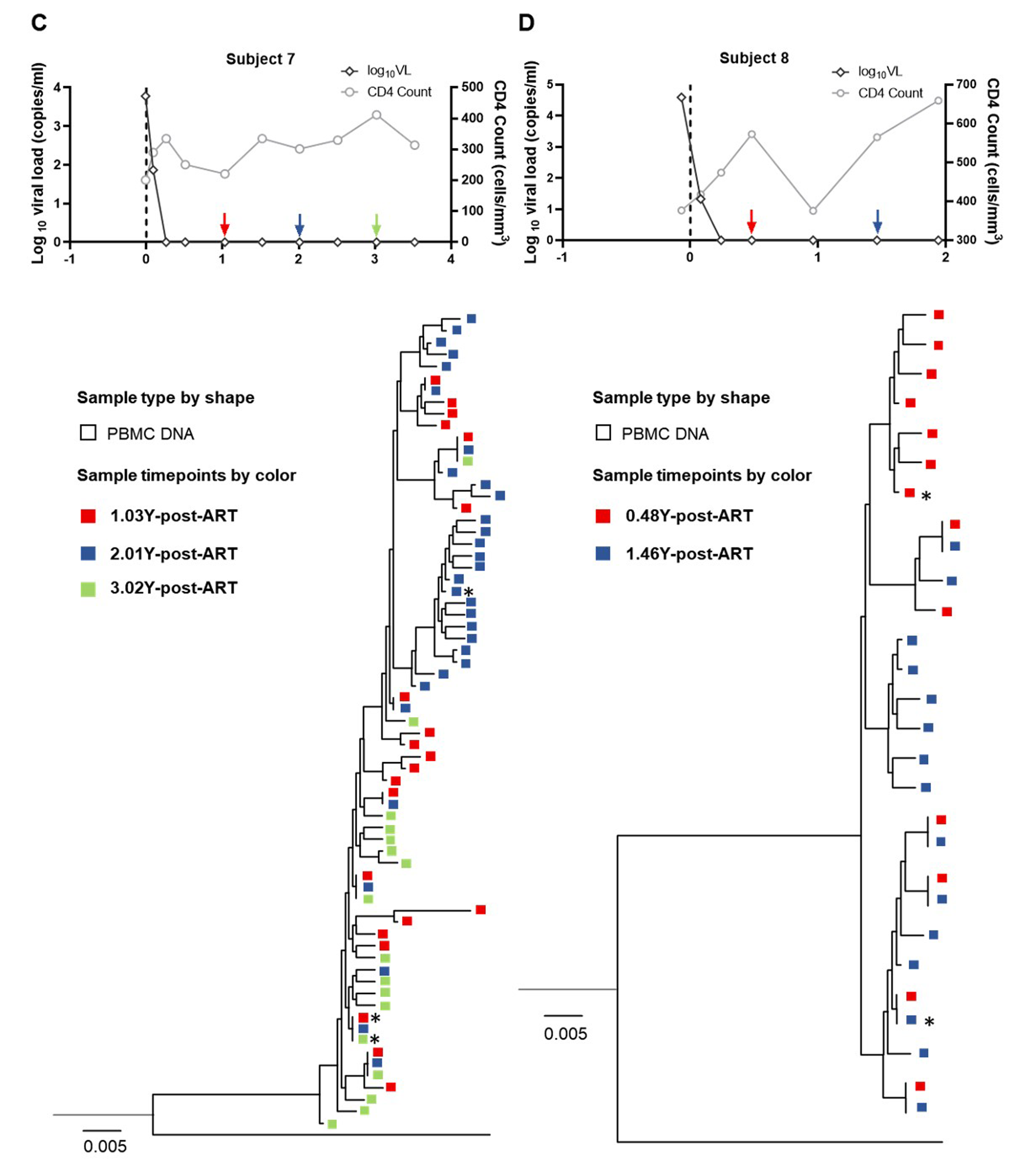

Supplement: Supplementary file 1 [file Data_Sheet_1.zip › Supplementary materials/Supplementary Figure S1-CD.tif]

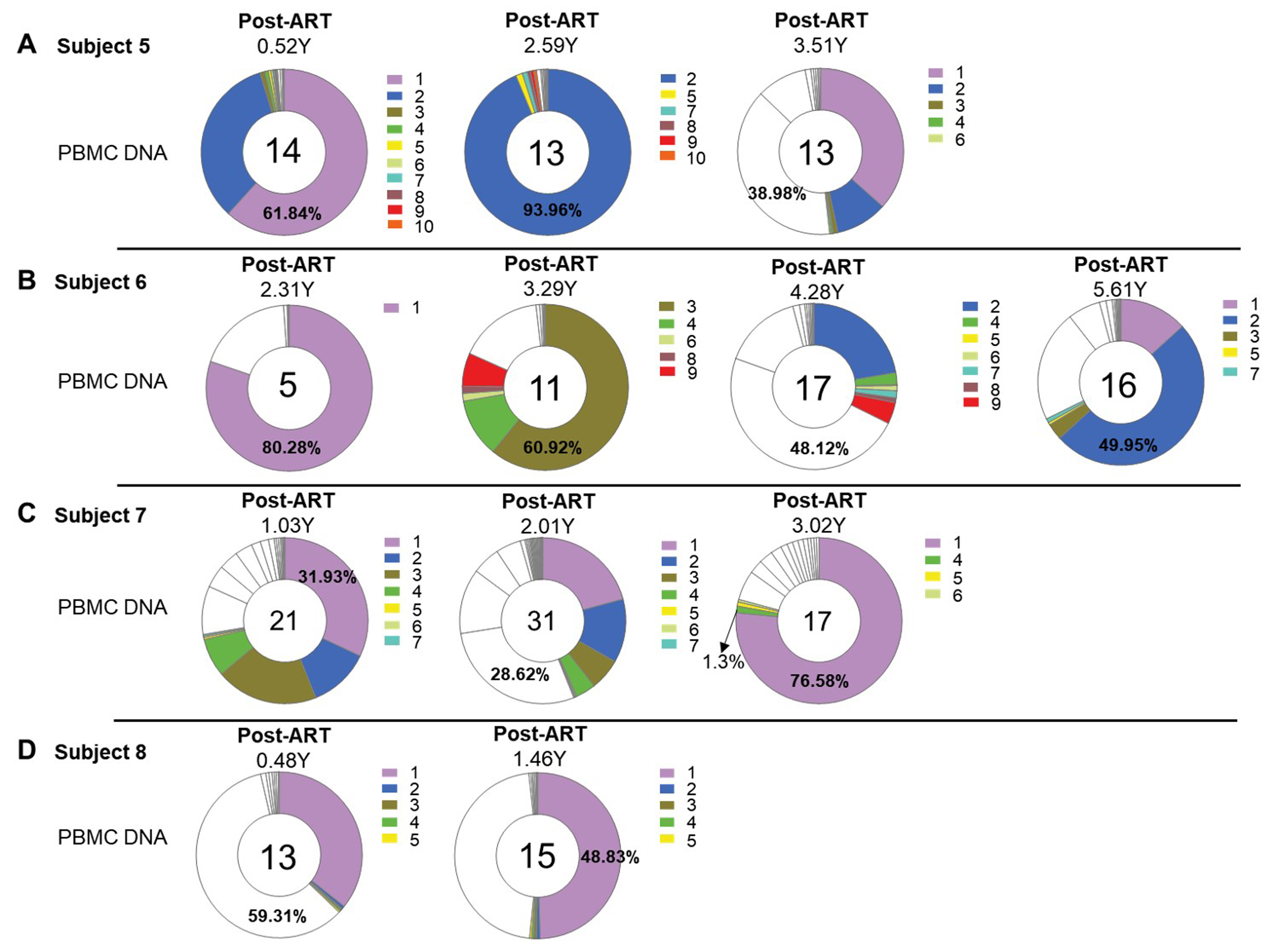

Supplement: Supplementary file 1 [file Data_Sheet_1.zip › Supplementary materials/Supplementary Figure S2.tif]

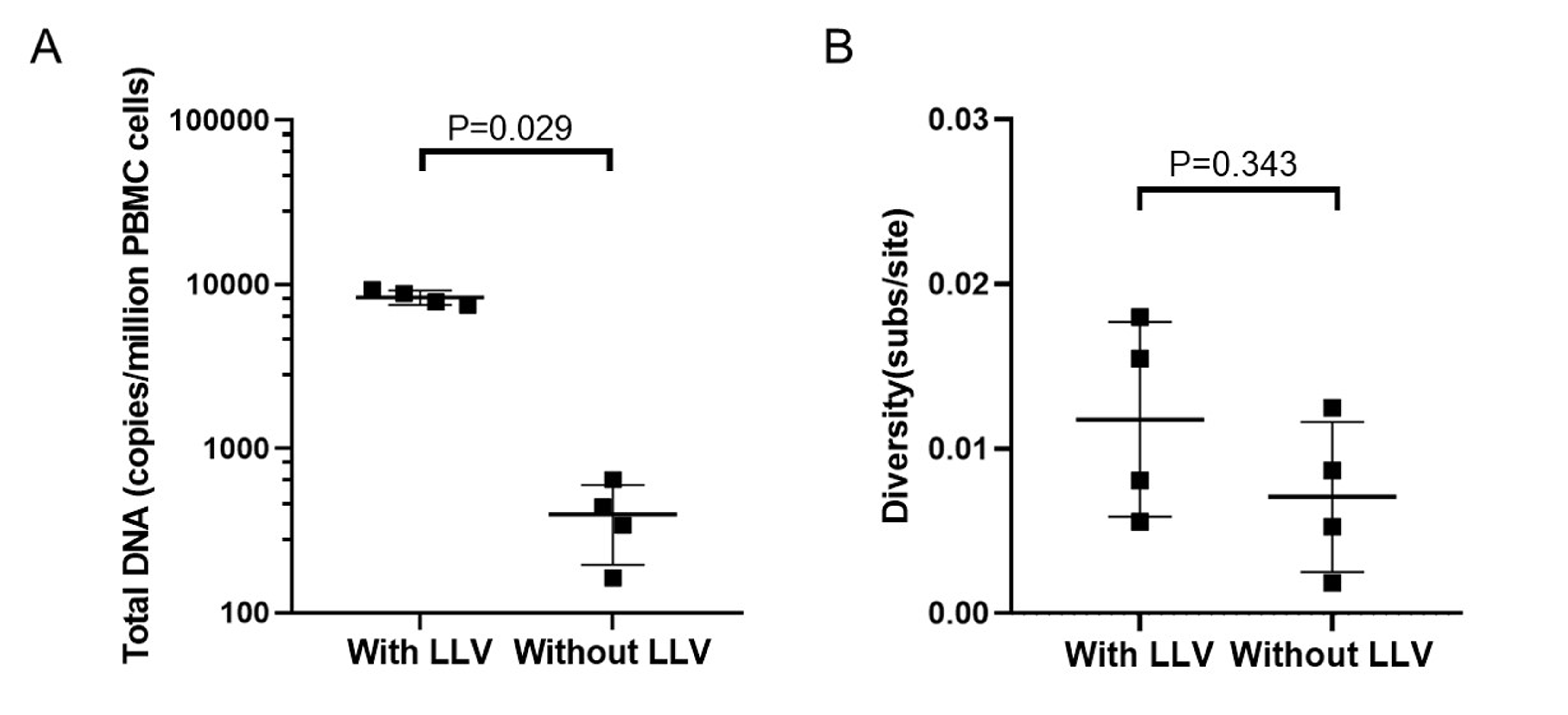

Supplement: Supplementary file 1 [file Data_Sheet_1.zip › Supplementary materials/Supplementary Figure S3.tif]
